# Health-Beneficial Phenolic Aldehyde in *Antigonon leptopus* Tea

**DOI:** 10.1093/ecam/nep041

**Published:** 2010-10-20

**Authors:** Vanisree Mulabagal, Ruby L. Alexander-Lindo, David L. DeWitt, Muraleedharan G. Nair

**Affiliations:** ^1^Bioactive Natural Products and Phytoceuticals, Department of Horticulture and National Food Safety and Toxicology Center, Michigan State University, East Lansing, MI, USA; ^2^Department of Basic Medical Sciences, The University of the West Indies, Mona, Kingston, Jamaica; ^3^Department of Biochemistry and Molecular Biology, Michigan State University, East Lansing, MI 48824, USA

## Abstract

Tea prepared from the aerial parts of *Antigonon leptopus* is used as a remedy for cold and pain relief in many countries. In this study, *A. leptopus* tea, prepared from the dried aerial parts, was evaluated for lipid peroxidation (LPO) and cyclooxygenase (COX-1 and COX-2) enzyme inhibitory activities. The tea as a dried extract inhibited LPO, COX-1 and COX-2 enzymes by 78%, 38% and 89%, respectively, at 100 *μ*g/mL. Bioassay-guided fractionation of the extract yielded a selective COX-2 enzyme inhibitory phenolic aldehyde, 2,3,4-trihydroxy benzaldehyde. Also, it showed LPO inhibitory activity by 68.3% at 6.25 *μ*g/mL. Therefore, we have studied other hydroxy benzaldehydes and their methoxy analogs for LPO, COX-1 and COX-2 enzymes inhibitory activities and found that compound **1** gave the highest COX-2 enzyme inhibitory activity as indicated by a 50% inhibitory concentration (IC_50_) at 9.7 *μ*g/mL. The analogs showed only marginal LPO activity at 6.25 *μ*g/mL. The hydroxy analogs **6**, **7** and **9** showed 55%, 61% and 43% of COX-2 inhibition at 100 *μ*g/mL. However, hydroxy benzaldehydes **3** and **12** showed selective COX-1 inhibition while compounds **4** and **10** gave little or no COX-2 enzyme inhibition at 100 *μ*g/mL. At the same concentration, compounds **14**, **21** and **22** inhibited COX-1 by 83, 85 and 70%, respectively. Similarly, compounds **18**, **19** and **23** inhibited COX-2 by 68%, 72% and 70%, at 100 *μ*g/mL. This is the first report on the isolation of compound **1** from *A. leptopus* tea with selective COX-2 enzyme and LPO inhibitory activities.

## 1. Introduction

The plant *Antigonon leptopus* is native to Mexico and commonly found in tropical Asia, Africa, the Caribbean and the Americas [1], and is one of the medicinal plants used in Jamaica. The hot tea prepared from the aerial portion of this plant is used traditionally for the prevention and treatment of cough and flu-related pain (2Mitchell and Ahmad, 2006 S. A. Mitchell and M. H. Ahmad, A review of medicinal plant research at the University of the West Indies, Jamaica, *West Indian Medicinal Journal* 55 [2] (2006), pages 243–253.). Studies have also shown that *A. leptopus* plant extracts exhibited anti-thrombin, analgesic, anti-inflammatory, anti-diabetic and lipid peroxidation inhibitory activities [2–5].

Phenolic compounds are widely distributed secondary metabolites in the plant kingdom and play an important role in their physiological and morphological functions [6]. These compounds are diverse group of phytochemicals derived from the shikimate and phenylpropanoid pathways in plants. Phenolic compounds are known as strong antioxidants and might prevent antioxidative damage to biomolecules such as DNA, lipids and proteins [7]. A number of epidemiological studies have shown that phenolic compounds can reduce the risk of chronic disorders such as cardiovascular disease and cancer [8]. Also, the phenolic compounds are reported to inhibit several stages of carcinogenesis *in vivo* [9]. In addition, they possess significant anti-inflammatory activity as suggested by both *in vitro* and *in vivo* studies [10].

Nonsteroidal anti-inflammatory drugs (NSAIDs) are the most popular products used for pain management. The NSAIDs act as inhibitors of prostaglandin synthesis by cyclooxygenase (COX) enzymes. The COX enzymes catalyze the conversion of arachidonic acid to prostaglandin endoperoxide, the immediate substrate, for a series of cell-specific prostaglandins, which play critical roles in various biological functions [8]. The two isoforms of COX differ mainly in their pattern of expression. COX-1 is expressed in most tissues and COX-2 is an inducible enzyme that is expressed in response to pro-inflammatory agents. Naturally occurring selective COX-2 inhibitors are significant since they can be consumed as supplements, reducing inflammation and potentially preventing cancer [11–14]. In the present study, we report the bioassay-guided isolation of a selective COX-2 inhibitor, a polyhydroxy benzaldehyde, from the tea extract and LPO and COX assay results for the tea extract of *A. leptopus*, the isolate and a number of its analogs.

## 2. Methods

### 2.1. Plant Material and Reagents

Aerial parts of *A. leptopus* were collected in Jamaica during 2005 and authenticated by Mr Patrick Lewis at University of the West Indies, Mona, Kingston, Jamaica. A voucher specimen (UWI 35294) has been deposited at the herbarium of the University of the West Indies, Mona, Kingston, Jamaica. The hydroxy benzaldehydes, 2-hydroxy benzaldehyde; 3-hydroxy benzaldehyde; 4-hydroxy benzaldehyde, 2,3-dihydroxy benzaldehyde; 2,4-dihydroxy benzaldehyde, 2,5-dihydroxy benzaldehyde, 3,4-dihydroxy benzaldehyde, 3,5-dihydroxy benzaldehyde, 2,4,5-trihydroxy benzaldehyde, 2,4,6-trihydroxy benzaldehyde and 3,4,5-trihydroxy benzaldehyde, were purchased from Sigma-Aldrich Chemical Co. (St Louis, MO).

### 2.2. Extraction and Isolation

*Antigonon leptopus* tea was prepared from the dried aerial parts of the plant (5 g) by soaking it with boiled water (50 mL) for 6 h. The resulting tea was evaporated under reduced pressure to yield a dark brown residue (606 mg). This residue was assayed LPO and COX enzyme inhibitory activities at 100 *μ*g/mL. This bioactive extract was then analyzed by TLC using CHCl_3_ and MeOH (4 : 1) as the mobile phase along with the methanolic extract prepared earlier in our laboratory [5]. The TLC profiles of tea residue and methanolic extract were similar except a new band observed under UV (*R*_*f*._  0.63). In order to isolate this UV-active compound, an aliquot of the water extract (200 mg) was purified by preparative TLC with CHCl_3_ and MeOH (4 : 1) as the developing solvents afforded compound **1** (0.7 mg). The structure of this compound was confirmed by ^1^H and ^13^C NMR spectral methods.

Compound **1**. ^1^H NMR (*d*_6_-DMSO): *δ* 9.83 (1H, s, CHO), 7.107 (1H, d, *J* = 8.4 Hz, H-6), 6.47 (1H, d, *J* = 8.4 Hz, H-5), 10.67, 10.32, 8.77 (each 1H, OH); ^13^C NMR (*d*_6_-DMSO): *δ* 193.1 (CHO), 153.2 (C-4), 150.9 (C-2), 132.2 (C-3), 123.7 (C-6), 115.4 (C-1), 108.3 (C-5). Therefore, the structure of compound **1** was established as 2,3,4-trihydroxy benzaldehyde and further confirmed by comparison of its NMR chemical shifts with its published spectral data [15].

### 2.3. Methylation of Hydroxy Benzaldehydes

Methylation of hydroxy benzaldehydes was performed according to published procedure [16]. The monohydroxy benzaldehyde (0.5 mmol) was dissolved in 5 mL of acetone in a round-bottomed flask. To this solution, anhydrous potassium carbonate (1 mmol) was added, sealed with septa and stirred for 10 min. Dimethyl sulfate (1 mmol) was then added to the reaction mixture by injection, stirred at room temperature for 6 h while monitoring the reaction by TLC for every 30 min. The reaction mixture was evaporated under vacuum and the residue dissolved in 25 mL of RO water, transferred to a separating funnel and extracted with ethyl acetate (25 mL), the ethyl acetate layer treated with saturated sodium bicarbonate solution and evaporated under vacuum. The product thus obtained was purified by preparative TLC using chloroform : methanol (80 : 20) as the mobile phase. The methylation of di- and trihydroxy benzaldehydes was carried out by using a similar procedure but with 2 and 3 mmols of potassium carbonate and dimethyl sulfate, respectively. The reaction times were 12 and 18 h for dihydroxy and trihydroxy benzaldehydes, respectively. The yields of mono-, di- and trimethoxy benzaldehydes were 90%, 86%, 75%, respectively. The compounds were characterized by NMR spectral methods. The NMR spectra of hydroxy benzaldehydes purchased from Sigma-Aldrich are not presented in this manuscript but confirmed that the compounds were pure.

Compound **13**. ^1^H NMR (*d*_6_-DMSO): *δ* 10.19 (1H, s, CHO), 7.51 (1H, d, *J* = 9.0 Hz, H-6), 7.00 (1H, d, *J* = 9.0 Hz, H-5), 3.98, 3.89, 3.89 (each 3H, s, OMe).

Compound **14**. ^1^H NMR (*d*_6_-DMSO): *δ* 10.35 (1H, s, CHO), 7.67 (1H, m, H-6), 7.22 (1H, d, *J* = 7.5 Hz, H-4), 7.09 (2, d, *J* = 7.5 Hz, H-3, 5), 3.90 (3H, s, OMe).

Compound **15**. ^1^H NMR (*d*_6_-DMSO): *δ* 9.98 (1H, s, CHO), 7.52 (2H, m, H-6, 5), 7.42 (1H, d, *J* = 1.5 Hz, H-2), 7.29 (1H, m, H-4), 3.83 (3H, s, OMe).

Compound **16**. ^1^H NMR (*d*_6_-DMSO): *δ* 9.87 (1H, s, CHO), 7.87 (2H, d, *J* = 7.0 Hz), 7.13 (2H, d, *J* = 7.0 Hz), 3.86 (3H, s, OMe).

Compound **17**. ^1^H NMR (*d*_6_-DMSO): *δ* 10.30 (1H, s, CHO), 7.40 (1H, dd, *J* = 1.8, 7.8 Hz, H-6), 7.26 (1H, dd, 1H, dd, *J* = 1.8, 7.8 Hz, H-4), 7.02 (1H, d, *J* = 7.8 Hz, H-5), 3.89, 3.98 (each 3H, s, OMe).

Compound **18**. ^1^H NMR (*d*_6_-DMSO): *δ* 10.17 (1H, s, CHO), 7.65 (1H, d, *J* = 8.7 Hz, H-6), 6.69 (1H, d, *J* = 2.4 Hz, H-3), 6.32 (1H, dd, *J* = 8.7, 2.4 Hz, H-5), 3.87, 3.90 (each 3H, s, OMe).

Compound **19**. ^1^H NMR (*d*_6_-DMSO): *δ* 10.34 (1H, s, CHO), 7.25 (1H, dd, *J* = 1.8, 9.0 Hz, H-6), 7.21 (1H, brs, H-5), 7.16 (1H, d, *J* = 9.0 Hz, H-4), 3.87, 3.98 (each 3H, s, OMe).

Compound **20**. ^1^H NMR (*d*_6_-DMSO): *δ* 9.9 (1H, s, CHO), 7.65 (1H, s, H-6), 7.55 (1H, s, H-2), 7.08 (1H, s, H-5), 3.8 (each 3H, s, 3, 4-OMe).

Compound **21**. ^1^H NMR (*d*_6_-DMSO): *δ* 9.91 (1H, s, CHO), 7.06 (2H, d, *J* = 2.4 Hz, H-2, 6), Compound **22**. ^1^H NMR (*d*_6_-DMSO): *δ* 10.19 (1H, s, CHO), 7.15 (1H, s, 6-H), 6.79 (1H, s, 3-H), 3.92, 3.91, 3.73 (each 3H, s, OMe).

Compound **23**. ^1^H NMR (*d*_6_-DMSO): *δ* 10.01 (1H, s, CHO), 6.14 (1H, s, H-3, 5), 3.85 (3H, s, 4-OMe), 3.98 (6H, s, 2.6-OMe).

Compound **24**. ^1^H NMR (*d*_6_-DMSO): *δ* 9.88 (1H, s, CHO), 7.26 (2H, s, H-2, 6), 3.86 (6H, s, 3, 5-OMe), 3.76 (3H, s, 4-OMe).

### 2.4. Cyclooxygenase Enzyme Inhibitory Assay

Cyclooxygenase enzyme inhibitory assay was carried out according to the published procedure [17]. The COX-1 enzyme was prepared from ram seminal vesicles purchased from Oxford Biomedical Research, Inc. (Oxford, MI). COX-2 enzyme was prepared from insect cell lysate diluted with Tris buffer (pH 7) to yield an approximate final concentration of 1.5 mg protein/mL. Activities of phenolic compounds were assessed by monitoring the initial rate of O_2_ uptake using a micro-oxygen chamber and electrode (Instech Laboratories, Plymouth Meetings, PA) attached to a YSI model 5300 biological oxygen monitor (Yellow springs Instrument, Inc., Yellow Springs, OH) at 37°C. Each assay mixture contained Tris buffer (0.6 mL, 0.1 M, pH 7), phenol (1 mM), hemoglobin (85 *μ*g) and DMSO or test samples (10 *μ*L). Cyclooxygenase enzymes (COX-1 or COX-2, 20 *μ*L) were added to the chamber and incubated for 3 min and the reaction was initiated by the addition of arachidonic acid (10 *μ*L of a 1 mg/mL solution). Analysis was performed in duplicate for each sample and the standard deviation was calculated for *n* = 2. The data were recorded using QuickLog for windows data acquisition and control software (Strawberry Tree, Inc., Sunnyvale, CA). The compounds **2**–**24** were tested at 100 *μ*g/mL concentration. The percent inhibition was calculated with respect to DMSO control. Compound **1** was tested at 25 *μ*g/mL and serial dilutions were made in order to obtain dose—response curve. Non-steroidal anti-inflammatory drugs, Aspirin (60 *μ*M), Celebrex (26 nM) and Vioxx (32 nM) were used as positive controls. Aspirin was purchased from Sigma-Aldrich Co. (St. Louis, MO) and Celebrex and Vioxx were physician samples kindly provided by Dr Subash Gupta, Sparrow Pain Center, MI, USA. We have used Vioxx as a positive control only in our *in vitro* assays. We continue to use it as a positive control in *in vitro* assays because Vioxx is a specific inhibitor of COX-2 enzyme among the non-steroidal anti-inflammatory agents (NSAIDs) produced and marketed. The use of Vioxx as a positive control in our *in vitro* assay is only for comparison purposes and not intended as a treatment. It is only used to study the mechanism of action of test compounds.

### 2.5. Lipid Peroxidation Inhibitory Assay

*In vitro* lipid peroxidation assay was carried out using large unilamellar vesicles (LUVs) using fluorescence spectroscopy according to the published procedure [17]. The phospholipid, 1-stearoyl-2-linoleoyl-sn-glycero-3-phosphatidylcholine (SLPC), in CHCl_3_ and fluorescence probe, 3-[*p*-(6-phenyl)-1,3,5-hexatrienyl] phenylpropionic acid (DPH-PA), in DMF (mg/mL) were homogenized and dried under reduced pressure. The LUVs were produced by suspension of the lipid-probe mixture (0.15 M NaCl, 0.1 mM EDTA and 0.01 M MOPS buffer maintained over Chelex resin) followed by ten freeze-thaw cycles in a dry ice-EtOH bath and extrusion (29 times) through a 100 nm pore-size membrane (Avestin Inc., Ottawa, Canada). The final assay volume was 2 mL, consisting of 100 *μ*L HEPES buffer (50 mM HEPES and 50 mM TRIS), 200 *μ*L 1 M NaCl, 1.64 mL of N_2_-purged water, 20 *μ*L of test sample or DMSO and 20 *μ*L of liposome suspension. Lipid peroxidation was initiated by the addition of 20 *μ*L of FeCl_2_ · 4H_2_O (0.5 mM) and the fluorescence was monitored at 0 min, 1 min, 3 min and every 3 min up to 21 min using a Turner Model 450 Digital Fluorometer. The decrease of relative fluorescence intensity over the time was used to determine the rate of peroxidation. The percentage of inhibition was calculated with respect to DMSO control. Extracts were tested at 100 *μ*g/mL. Pure compounds were tested at 6.25 *μ*g/mL since the activity of compound **1** was >50%. Commercial antioxidants BHA (butylated hydroxyanisol), BHT (butylated hydroxytoluene) and TBHQ (t-butyl hydroquinone) were tested at 1 *μ*g/mL.

## 3. Results

### 3.1. Bioactive Constituents in *A. leptopus* Tea

**Figure 1:**
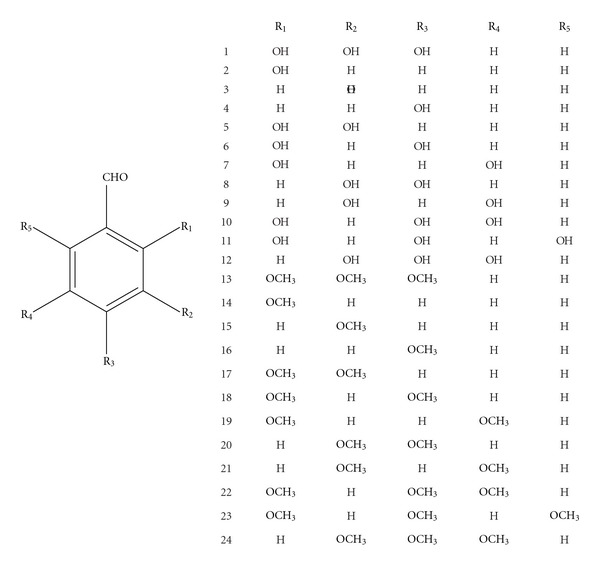
Structures of hydroxy and methoxy benzaldehydes.

**Figure 2:**
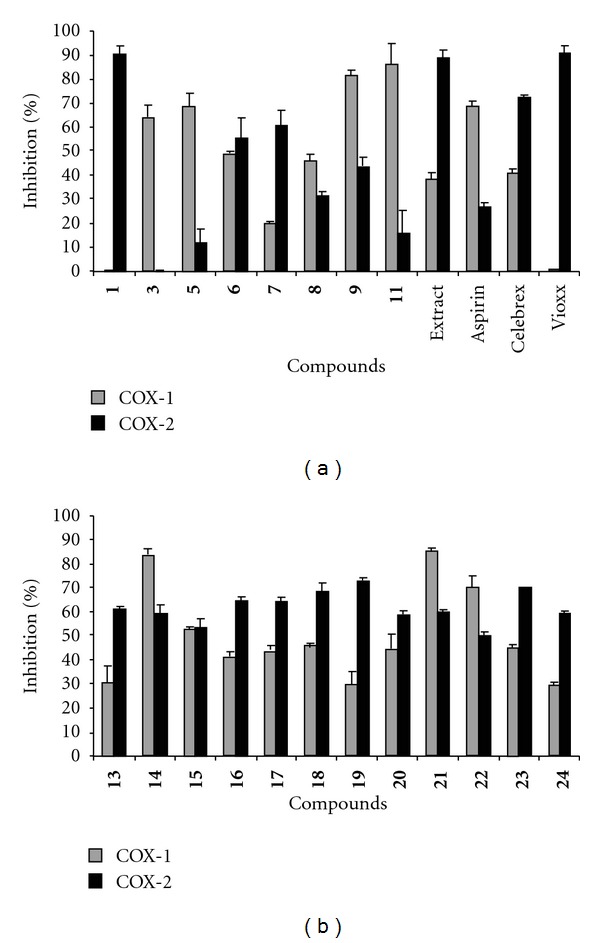
*In vitro* COX-1 and COX-2 enzyme inhibitory activities of: (a) hydroxyl benzaldehydes and (b) methoxy benzaldehydes. The concentration of compound **1** tested in this assay was 25 and the tea extract and compounds **2**–**24** at 100 *μ*g/mL. Positive controls used in the assay were Aspirin (60 *μ*M), and Celebrex (26 nM) and Vioxx (32 nM). DMSO was used as solvent control and the percent inhibition was calculated with respect to DMSO control. Vertical bars represent average of two experiments ± SD.

The crude water extract obtained after evaporating the tea gave strong LPO, COX-1 and COX-2 enzyme inhibitory activities as indicated by 78%, 38.3% and 89%, respectively, at 100 *μ*g/mL. The bioassay-guided isolation of this extract gave a phenolic compound with selective COX-2 enzyme inhibitory activity. The structure of compound **1** was identified to be 2,3,4-trihydroxy benzaldehyde [1] by using NMR spectral data and further confirmed by TLC comparison with an authentic sample (Figure 1). It inhibited COX-2 enzyme by 90% and was inactive against COX-1 enzyme at 25 *μ*g/mL (Figure 2(a)). Serial dilutions of compound **1** were made to determine the 50% inhibitory concentration. Therefore, the dose-dependent evaluation of the inhibitory activity of compound **1** against COX-2 enzyme gave the 50% (IC_50_) inhibitory concentration as 9.7 *μ*g/mL. Interestingly, compound **1** did not show any activity against COX-1 enzyme at 500 *μ*g/mL, the highest concentration tested.

### 3.2. Activity of Methoxylated Benzaldehydes

Although compound **1** was isolated from *A. leptopus* tea as a natural product, the synthetic version of it is available in the market along with a number of other substituted hydroxyl benzaldehydes. Studies on antioxidant and anti-inflammatory activity of some of the hydroxy benzaldehydes and their analogs have been reported in the literature [18, 19]. Therefore, we have compared the structure-activity relationship of some of the commercially available hydroxy benzaldehydes (**2**–**12**) with compound **1** along with their methoxy derivatives (**13**–**24**) using *in vitro* LPO and COX enzyme inhibitory assays. The hydroxyl benzaldehydes were methylated in our laboratory to yield corresponding methoxy derivatives [15]. All compounds were characterized by proton and carbon NMR experiments (Figure 1).

Among the monohydroxy tested, compound **3** selectively inhibited COX-1 by 64% at 100 *μ*g/mL. The inhibitory values of compounds **14** and **15** against COX-1 and COX-2 enzymes were 83% and 53% and 59% and 53%, respectively at 100 *μ*g/mL (Figure 2(b)). Although compound **4** showed little or no activity against COX enzymes, the methylated product **16** inhibited COX-1 and COX-2 enzymes by 41% and 65%, respectively, at 100 *μ*g/mL (Figure 2(a)).

Among the dihydroxy benzaldehydes, compound **7** was the most active against COX-2 enzyme with an inhibition of 61% at 100 *μ*g/mL. Similarly, compounds **6** and **9** showed moderate inhibition against COX-2 enzyme by 55% and 43%, respectively. However, compounds **5** and **9** inhibited COX-1 enzyme by 68% and 82%, respectively, at the same concentration (Figure 2(a)). The COX-2 enzyme inhibitory activity of compound **5** was increased to 64% after methylation as shown in Figure 2(a). Other methylated compounds, **18**–**21**, showed little or no variation in the activity profile. The inhibitory values observed for compounds **18**, **19**, **20** and **21** against COX-1 and COX-2 enzymes were 46%, 30%, 44%, 85% and 68%, 72%, 59%, 60%, respectively, at 100 *μ*g/mL (Figure 2(b)).

Among the trihydroxy benzaldehydes tested, compound **10** was not inhibitory to COX-1 and COX-2 enzymes when tested at 100 *μ*g/mL. However, its methylated product **22** inhibited COX-1 and COX-2 enzymes by 70% and 50%, respectively, at the same concentration (Figure 2(b)). This is evident from the activity profile of hydroxy benzaldehyde **11**, a reversal of COX-1 and COX-2 activities due to methylation from 86 and 16% at 100 *μ*g/mL to 45% and 70%. Commercial standards Vioxx, Celebrex and aspirin were tested at 32 nM, 26 nM and 60 *μ*M concentrations. The inhibition values of Aspirin and Celebrex against COX-1 annd COX-2 enzymes were 69%, 41%, 27% and 72%, respectively. Vioxx is inactive to COX-1 and inhibited COX-2 enzyme by 91% (Figure 2(a)). The varying concentrations of positive controls used were necessary to keep the COX enzyme inhibition between 50% and 100%.

**Figure 3:**
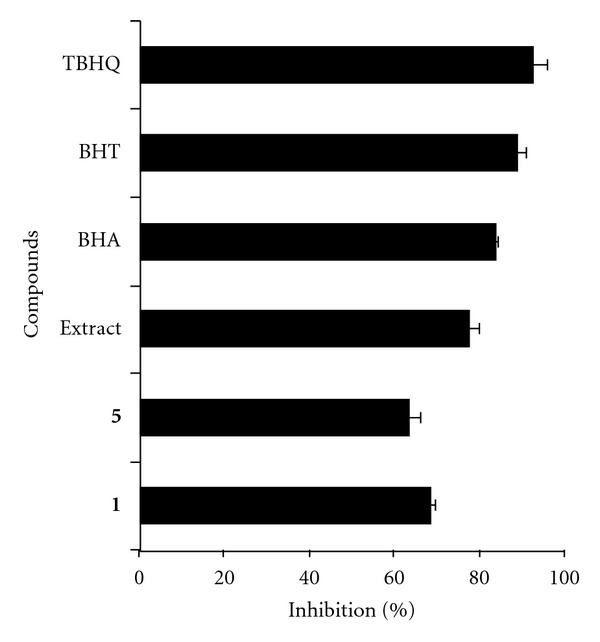
Lipid peroxidation inhibitory activities of tea prepared from *A. leptopus* and compounds **1** and **5**. Positive controls, antioxidants BHA (butylated hydroxyanisol), BHT (butylated hydroxytoluene) and TBHQ (t-butyl hydroquinone) were tested at 1 *μ*g/mL. Extract and compounds were tested 100, 6.25 *μ*g/mL, respectively. Water or DMSO was used as solvent control and the percent inhibition was calculated with respect to water or DMSO control. Vertical bars represent the average of two experiments ± SD.

The LPO assay revealed that compounds **1** and **5** were the most active and inhibited LPO by 68% and 64%, respectively, at 6.25 *μ*g/mL (Figure 3). The LPO inhibition observed for compounds **2**, **6** and **8** was in the range of 40%–45% at the same concentration (data not shown). The methoxy benzaldehydes showed little or no activity against LPO when tested at 6.25 *μ*g/mL. This is in agreement with the general understanding that hydroxy group is essential for antioxidant activity and methylation may result in lower activity [15]. Standard antioxidants BHA, BHT and TBHQ were used as positive controls in the assay and the inhibition values were in the range of 84%–93% for these compounds at 1 *μ*g/mL (Figure 3).

## 4. Discussion

Traditional use of the tea prepared from the aerial parts of *A. leptopus* has been implicated to the alleviation of swelling and cold. Studies have shown that the anti-inflammatory activity of *A. leptopus* plant material was due to the presence of phenolic compounds present in the organic extract [4, 5]. However, the constituents of the tea, the form consumed as a remedy, and its biological activity have not been studied till now. The results from the current study suggest that several compounds present in the tea extract also exist in the organic extract. The tea contained the phenolic compound 2,3,4-trihydroxy benzaldehyde with selective COX-2 enzyme inhibitory activity.

Phenolic compounds have attracted considerable interest in recent years due to their potential health benefits such as anti-oxidant, antimicrobial, anti-inflammatory and cardioprotective activities [21–24]. The inhibition of prostaglandin synthesis by phenolic compounds has also been reported [25–27] and their COX inhibitory activity was correlated to the antioxidant properties [27]. This observation is evident from our COX assay results that benzaldehyde is inactive but its hydroxylated analogs showed varying degrees of COX-1 and COX-2 enzyme inhibitory activities, although a trend in activity related to the number of hydroxy and methoxy substitutions were not evident.

The amount of aerial portions of *A. leptopus* used in the preparation of tea and the frequency of its consumption by folklore is unclear. Although the amount of *A. leptopus* plant material used in the preparation of tea in our study was primarily to yield adequate amount of extract for bioassay and the isolation of bioactive principles, it is evident that a tea prepared from 5 g of dried plant material deliver about 2.1 mg of COX-2 active hydroxy benzaldehyde, compound **1**. This is in comparison to the efficacy of Vioxx observed in our *in vitro* bioassay. Therefore, a 50% inhibitory concentration (IC_50_) of 9.7 *μ*g/mL observed for compound **1** is in par with the 32 nM concentration of Vioxx. Hypothetically, the consumption of *A. leptopus* tea containing 650–700 mg of extract containing compound **1** per day could yield COX-2-related pain relief similar to the daily dose of an NSAID to a person with an average body weight of 70 kg. In conclusion, our efficacy results of the tea and active components present in it support the use of *A. leptopus* tea as an ethnomedicine to ameliorate inflammatory pain and may be beneficial to improve the quality of life.
